# Riddell on the Constitution of Matter

**Published:** 1846-04

**Authors:** 


### RIDDELL ON THE CONSTITUTION OF MATTER.

This is the subject of a learned article in the New Orleans Journal of Medicine, for March, which has been issued in pamphlet form. It is not a subject to interest the majority of readers, but a few, it is to be hoped, will meet with it, who can do justice to the recondite speculations of the author. He has ventured to set up opinions at variance with some of the received doctrines of natural science, and sustains them by an imposing array of arguments, but whether conclusively or not we leave to others more versed in mathematical science than ourselves to decide. The conclusion of Dr. Riddell's paper is beautiful, and we take great pleasure in quoting it:

"Deeply impressed with the conviction, that all the current physical phenomena of the universe are but rational and regular sequences from rational and regular causes, I have discarded the idea of occult and inherent qualities. Where the relation of things seams occult and incomprehensible, I have uniformly ventured to infer that they merely seem so, because the true and essential relations have not yet been developed. When we look abroad into the workings of nature, few, comparatively, are the physical objects and relations which we truly comprehend: and countless, those which to us are dim and distant forms; admonishing us of our weakness, and of our infinitely small importance in the grand system of the universe. But let us not despair. For since we see that neither matter nor motion admits of natural annihilation, but that both possess the passive attributes of endless perpetuity; let us exult in the firm conviction, which by parity of reason forces itself upon us, that the gift of the Divine Being also,—that more subtle portion of ourselves which thinks, remembers and reasons, is destined to immortality."

